# Recent Design and Application Advances in Micro-Electro-Mechanical System (MEMS) Electromagnetic Actuators

**DOI:** 10.3390/mi16060670

**Published:** 2025-05-31

**Authors:** Jianqun Cheng, Ning Xue, Bocang Qiu, Boqi Qin, Qingchun Zhao, Gang Fang, Zhihui Yao, Wenyi Zhou, Xuguang Sun

**Affiliations:** School of Electronics and Communication Engineering, Quanzhou University of Information Engineering, Quanzhou 362000, China; jcheng@qzuie.edu.cn (J.C.);

**Keywords:** electromagnetic actuation, actuator integration, micro-electro-mechanical systems

## Abstract

Micro-electro-mechanical system (MEMS) electromagnetic actuators have rapidly evolved into critical components of various microscale applications, offering significant advantages including precision, controllability, high force density, and rapid responsiveness. Recent advancements in actuator design, fabrication methodologies, smart control integration, and emerging application domains have significantly broadened their capabilities and practical applications. This comprehensive review systematically analyzes the recent developments in MEMS electromagnetic actuators, highlighting core operating principles such as Lorentz force and magnetic attraction/repulsion mechanisms and examining state-of-the-art fabrication technologies, such as advanced microfabrication techniques, additive manufacturing, and innovative material applications. Additionally, we provide an in-depth discussion on recent enhancements in actuator performance through smart and adaptive integration strategies, focusing on improved reliability, accuracy, and dynamic responsiveness. Emerging application fields, particularly micro-optical systems, microrobotics, precision micromanipulation, and microfluidic components, are extensively explored, demonstrating how recent innovations have significantly impacted these sectors. Finally, critical challenges, including miniaturization constraints, integration complexities, power efficiency, and reliability issues, are identified, alongside a prospective outlook outlining promising future research directions. This review aims to serve as an authoritative resource, fostering further innovation and technological advancement in MEMS actuators and related interdisciplinary fields.

## 1. Introduction

Micro-electro-mechanical systems (MEMS) technology has profoundly transformed various engineering and technological fields by enabling unprecedented miniaturization and the integration of mechanical components, sensors, actuators, and electronic circuits onto compact microscale platforms [1]. This transformation has facilitated the emergence and widespread adoption of MEMS devices across diverse applications, including consumer electronics, biomedical instrumentation, automotive systems, microrobotics, telecommunications, and aerospace engineering. Among these components, MEMS actuators play a particularly critical role, providing essential functionalities such as precise force generation, accurate motion control, and meticulous positioning capabilities. These functionalities are fundamental for sophisticated operations required in advanced biomedical devices, autonomous robotics, microfluidic systems, and precision optical instrumentation [2,3,4].

Compared with the traditional mechanical driver, MEMS actuators are inherently miniaturized and highly compact, featuring microscale designs that minimize power consumption and allow seamless integration with CMOS circuits. MEMS actuators are broadly categorized into electrostatic, piezoelectric, thermal, and electromagnetic actuators, each leveraging distinct physical principles to achieve specific functional advantages. Within this landscape, electromagnetic actuators have drawn significant attention due to several inherent advantages such as a higher force density, increased displacement range, swift response times, and lower driving voltages. Unlike electrostatic actuators and piezoelectric actuators, which require high voltages to achieve significant force, electromagnetic actuators can generate substantial forces at low driving voltages typically within a few volts. This characteristic makes them ideal for applications demanding robust mechanical output, such as microfluidic pumps, microvalves, and optical scanning mirrors [5,6]. A detailed comparison is shown in Table 1. The inductive nature of electromagnetic actuation allows for rapid changes in force application, enabling high-frequency operation with minimal lag. This outperforms thermal actuators, which suffer from slow heating and cooling cycles, limiting their switching speed.

However, MEMS electromagnetic actuators have encountered substantial barriers related to their complex fabrication processes, challenges in integrating magnetic materials, relatively higher power consumption, and scalability limitations. Recently, significant advancements have been made to address these issues through innovative fabrication techniques and materials science breakthroughs. Novel microfabrication methods such as lithography, electroplating, molding, hybrid micromachining processes, and additive manufacturing (3D printing) techniques have enabled more straightforward, reliable, and cost-effective fabrication pathways. Additionally, advancements in soft magnetic materials, magnetic nanocomposites, and flexible substrates have considerably enhanced the actuators’ performance, facilitating better integration within microsystems and broadening their application scope [7,8]. Moreover, concurrent developments in intelligent actuator control strategies and integrated sensor–actuator feedback mechanisms have significantly elevated the precision, adaptability, and reliability of MEMS electromagnetic actuators. These advances have opened new avenues for applications requiring dynamic responsiveness and intricate maneuvering capabilities, such as microrobotics for minimally invasive medical procedures, high-precision microfluidic manipulation for lab-on-chip systems, adaptive micro-optical components in imaging systems, and microscale energy harvesters for autonomous systems [9,10].

**Figure 1:**
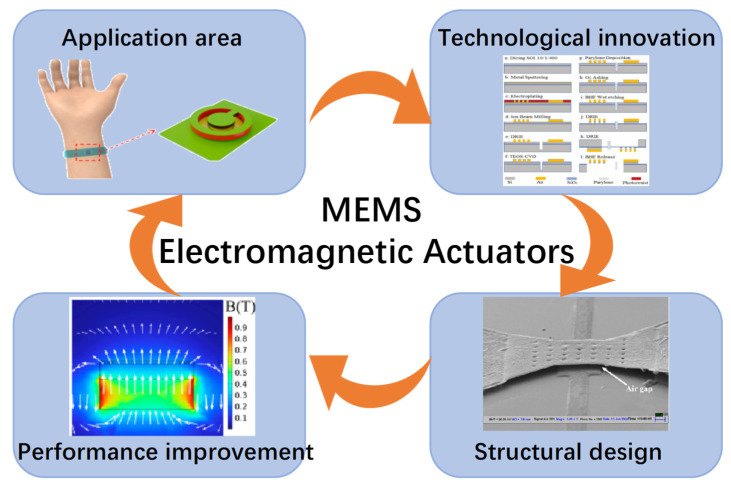
Overview of MEMS electromagnetic actuators.

As shown in Figure 1, considering these recent significant advances and the expanding application horizon, a critical and comprehensive review of MEMS electromagnetic actuators is both timely and essential. This review aims to present an exhaustive analysis of recent innovations, detailed discussions of state-of-the-art fabrication methodologies, in-depth considerations of actuator design criteria, and a systematic exploration of current and potential applications. By categorizing MEMS electromagnetic actuators based on fundamental operating principles, examining recent advancements in fabrication technology, and highlighting their successful integration into practical applications, this review intends to provide an authoritative reference for researchers and industry professionals [11].

In addition, this review will not only articulate the current state-of-the-art but also identify key challenges and propose future research directions that can drive further innovation and adoption of MEMS electromagnetic actuators. We hope this comprehensive exploration will serve as a valuable resource and guidance tool, fostering informed decision-making and strategic development in MEMS, microsystem design, and related interdisciplinary fields.

## 2. MEMS Electromagnetic Actuator Designs

Electromagnetic actuation principles have witnessed increasing adoption within MEMS owing to their intrinsic advantages, including their notably high force densities, rapid response capabilities, low operational voltages, and favorable scalability. These unique attributes position electromagnetic actuators as particularly suitable candidates for diverse microsystem applications, such as micro-optical systems, microrobotics, biomedical microdevices, microfluidics, and microscale energy-harvesting devices. In contrast to other actuation modalities such as electrostatic, piezoelectric, and thermal mechanisms, electromagnetic actuation leverages interactions between magnetic fields and electric currents, enabling robust and reliable actuator operations even under stringent system constraints.

Based upon the fundamental electromagnetic phenomena underlying their operation, MEMS electromagnetic actuators are commonly classified into three primary categories: Lorentz force actuators and magnetic attraction or repulsion actuators. Each of these categories exploits distinct electromagnetic interactions to achieve targeted actuator behaviors suitable for specific microdevice requirements. Lorentz force actuators operate on the principle of the Lorentz force, which describes the force exerted on a current-carrying conductor placed within a magnetic field. By precisely controlling electric currents and magnetic fields at microscale dimensions, Lorentz force actuators provide highly accurate, predictable, and linear actuation responses. Due to their capability to produce relatively large displacement and their rapid response time with excellent repeatability, these actuators have been extensively adopted in microsystems requiring precise motion control, such as micro-scanners, micropositioning stages, and optical switching devices [12].

Magnetic attraction or repulsion actuators exploit the inherent forces between permanent magnets or between magnets and soft magnetic materials. Unlike Lorentz force actuators, these actuators rely predominantly on static or semi-static magnetic fields and magnetic permeability contrasts rather than dynamic current flow alone. By harnessing magnetic attraction and repulsion phenomena, such actuators achieve substantial force outputs with lower power consumption. Typical applications include microvalves, micro-pumps, switches, and various microrobotic systems that benefit significantly from high force generation at minimal power and reduced system complexity [13,14].

Beyond the fundamental actuation mechanisms, actuator performance and application specificity are strongly influenced by precise design configurations and geometric parameters. The effectiveness of electromagnetic MEMS actuators is closely correlated to the optimized spatial arrangement of coils, magnets, magnetic circuits, and flux-guiding components. Geometric parameters such as the coil dimensions, magnetic core shapes, magnetic flux path designs, and spacing critically determine the actuator’s force magnitude, stroke range, energy efficiency, and thermal management capabilities. As a result, considerable research attention has been directed towards developing optimized design methodologies, computational modeling techniques, and simulation-driven design optimization procedures, thus ensuring maximum actuator performance tailored to particular microdevice applications [15,16].

This chapter presents a comprehensive analysis and synthesis of Lorentz force and magnetic attraction/repulsion MEMS actuators, alongside discussions of typical actuator design configurations, geometrical influences, and their consequent implications for device performance.

### 2.1. Lorentz Force Actuators

**Figure 2:**
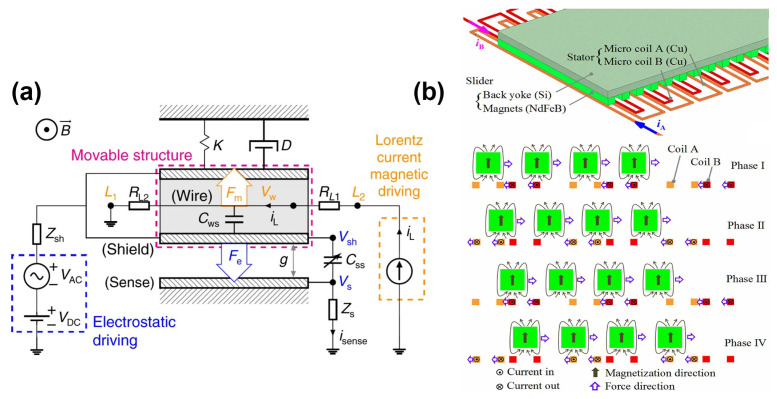
(**a**) Principle of operation of a shielded Lorentz-force magnetometer [26]. (**b**) Actuation principle depicted in cross-sectional view of the magnetic attraction/repulsion MEMS actuator [35]. Reprinted/adapted with permission from Ref. [35]. 2025, IOP Publishing Ltd.

Lorentz force actuators constitute one of the predominant and extensively adopted categories of MEMS electromagnetic actuators. Their fundamental operational principle is based upon the Lorentz force, which arises when an electric current passes through a conductor within a magnetic field, generating a mechanical force perpendicular to both the current flow and the magnetic field direction as shown in Figure 2a. Due to their inherent linear response characteristics, rapid actuation capabilities, substantial force density, and excellent scalability, Lorentz force actuators have become integral components across numerous MEMS applications, particularly those demanding precision positioning, rapid response, and reliable operational stability [17,18]. Typically, MEMS-based Lorentz force actuators consist of microfabricated conductive elements, such as suspended coils or planar conductor patterns, strategically positioned within stationary external magnetic fields provided by permanent magnets or integrated magnetic thin-films. Upon the application of an electrical current, the resultant Lorentz force induces mechanical displacement, rotation, or vibration. Precise actuation control is conveniently realized by modulating the magnitude and polarity of the input current, rendering Lorentz force actuators particularly suitable for dynamic, high-performance microactuation applications including optical MEMS scanning mirrors, microscale positioning stages, biomedical manipulators, and microfluidic control components [19,20].

The performance and efficacy of Lorentz force actuators significantly depend upon several key design factors. The conductor geometry, including the coil dimensions and the number of turns, directly influences the magnitude of the achievable actuation force. While increasing the conductor length or coil density generally enhances actuator force, these design adjustments must be carefully balanced against increased electrical resistance, Joule heating, and associated thermal management challenges. Additionally, the strength and uniformity of the magnetic field are crucial determinants of actuator consistency and efficiency, emphasizing the importance of the strategic selection and placement of permanent or electromagnets during the design phase [21,22,23].

Thermal considerations are also critical in Lorentz force actuator design. Continuous current flow inherently generates heat, potentially leading to thermal expansion, material fatigue, and performance degradation. To mitigate these issues, MEMS actuator designs frequently employ thermally conductive substrates, optimized conductor structures, and integrated heat dissipation pathways.

**Figure 3:**
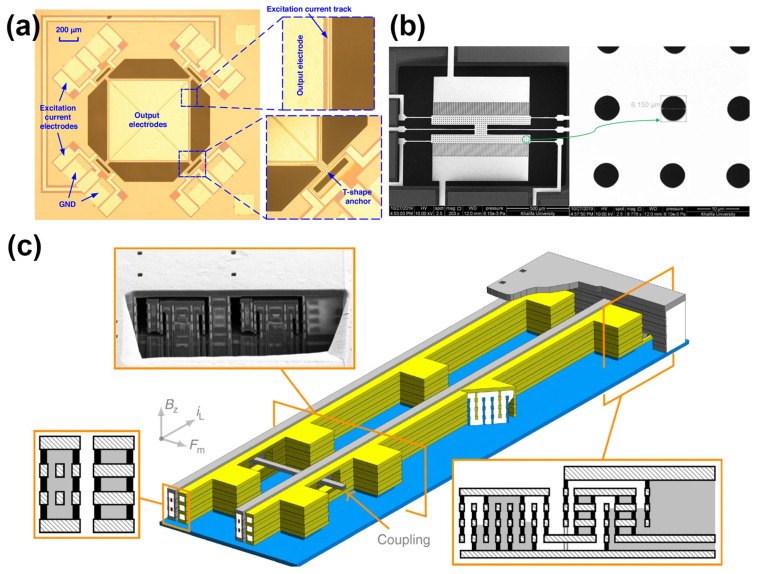
(**a**) Optical micrograph of the fabricated lorentz force magnetometer which comprises a square-plate AlN-on-Si resonator with a side length of 800 µm [24]. (**b**) SEM images of the fabricated device with a closeup on one the proof mass perforations [25]. (**c**) 3D perspective of the z magnetometer: lateral resonance. Arrows indicate direction of sensed magnetic field, magnetic force, and Lorentz current. Cross-sections and SEM images for each device are shown [26].

Tu et al. proposed a single-structure three-axis Lorentz force magnetometer based on an AlN-on-Si MEMS resonator, where a precisely engineered current path and multimode vibration control enabled efficient detection of tri-axial magnetic fields within a compact device architecture, as shown in Figure 3a [24]. The device integrates out-of-plane drumhead modes and in-plane extensional modes to sense x/y-axis and z-axis magnetic fields, respectively, demonstrating excellent axis decoupling performance. Furthermore, the authors introduced, for the first time, an equivalent electrical model tailored for this type of piezoelectric LFM, which effectively isolates the Lorentz force response from parasitic capacitive interference. Compared to conventional capacitive-based designs, this device features atmospheric-pressure operation, structural simplicity, high sensitivity (up to 6.75 ppm/mT for the z-axis), and notable stability and low power consumption. These attributes indicate its strong potential for multi-degree-of-freedom magnetic field sensing and integration in inertial navigation systems and other advanced applications. Mohammed et al. proposed a novel MEMS Lorentz force magnetometer featuring a longitudinal capacitive transducer to overcome the sensitivity limitations inherent in traditional transverse designs, as shown in Figure 3b. Unlike conventional approaches that suffer from squeeze-film damping and limited geometric scalability, their architecture employs longitudinal comb fingers, enabling enhanced sensitivity by increasing finger count without compromising quality factor. Fabricated using a low-cost SOI MEMS process, the device demonstrates a high Q-factor of 200 under near-vacuum conditions, static capacitance of 1.27 pF. Simulation and experimental results confirm the effectiveness of the design. This work highlights a promising path for integrating high-sensitivity, low-power magnetometers in space-constrained applications such as nanosatellites and low Earth orbit missions [25]. Valle et al. present CMOS-MEMS Lorentz-force magnetometers and discuss the challenges, modeling approaches, and design solutions for achieving high-yield CMOS-MEMS devices, as shown in Figure 3c. The magnetometers were packaged, characterized, and subjected to reliability tests, demonstrating competitive performance compared to commercial magnetometers. The paper also analyzes the sensitivity and offsets induced by the Lorentz current and proposes solutions to mitigate the electrical and electrostatic interference issues that are common in Lorentz-force magnetometers [26].

### 2.2. Magnetic Attraction/Repulsion Actuators

Magnetic attraction/repulsion actuators represent a prominent category within MEMS electromagnetic actuator technologies, relying fundamentally on magnetic field interactions between magnetized components. Unlike Lorentz force actuators, which generate actuation forces through current-carrying conductors immersed within magnetic fields, magnetic attraction and repulsion actuators leverage either the attractive or repulsive forces emerging from interacting magnetic elements, such as permanent magnets or electromagnets integrated into microstructures. Due to their straightforward operational mechanisms, strong force densities, and capability for non-contact actuation, these actuators have garnered significant research interest and practical adoption in numerous microscale applications [27,28,29].

The operational principles underlying magnetic attraction/repulsion actuators are governed primarily by the interactions between magnetic dipoles. These interactions generate forces that vary according to the strength, orientation, separation, and magnetic permeability of the interacting elements. Attractive actuators typically utilize opposite polarities to generate pulling forces that decrease rapidly with increasing distance, thereby enabling precise position control and stable positioning as shown in Figure 2b. Conversely, repulsive actuators employ like polarities to produce pushing forces, useful in applications demanding separation or levitation. The mathematical description of these interactions is commonly derived from Maxwell’s equations, which quantify force magnitudes based on magnetic field gradients, magnetization characteristics, and spatial configurations [30,31].

Magnetic attraction/repulsion MEMS actuator performance depends heavily on the magnetic alignment precision, field strength, and gap control. Achieving high actuation forces requires carefully engineered microstructures with minimal magnetic reluctance paths and strategically arranged magnetic elements [32]. Furthermore, the geometry and layout of the magnets significantly influence the actuator’s dynamic response, force linearity, and operational range. Thus, careful attention to magnet orientation, spacing, and field uniformity is essential in actuator design optimization.

**Figure 4:**
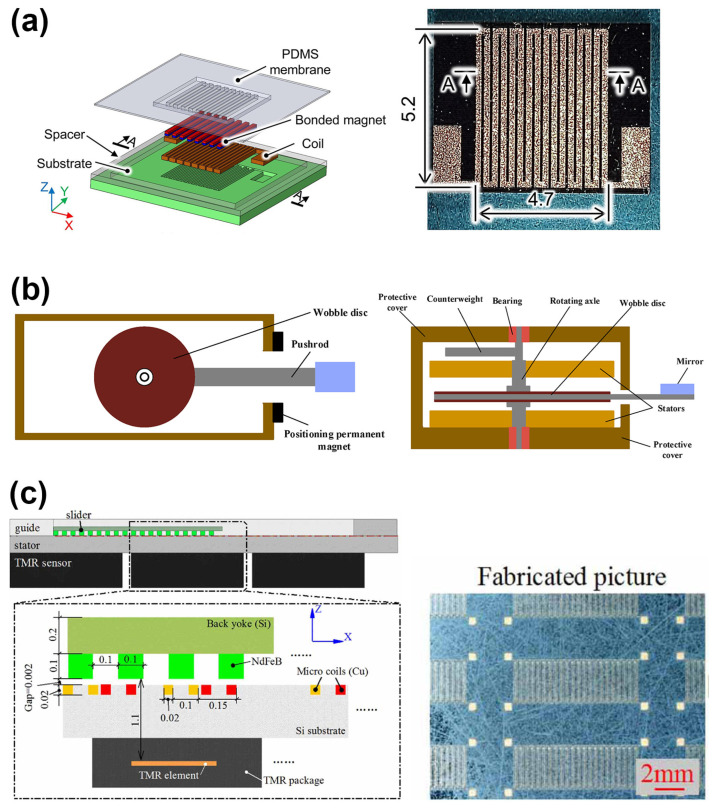
(**a**) Proposed EMMA utilizing molded bonded magnets and a fabricated meandering planar coil [33]. Reprinted/adapted with permission from Ref. [33]. 2025, Elsevier. (**b**) The structure diagram of the swing-type electromagnetic actuator [34]. (**c**) Cross-sectional view of the microactuator [35]. Reprinted/adapted with permission from Ref. [35]. 2025, IOP Publishing Ltd.

Qi et al. develop a MEMS-based electromagnetic membrane actuator (EMMA) that utilizes bonded magnets with a large displacement shown in Figure 4a [33]. They proposed fabricating the bonded magnets using micro compression molding with a soft PDMS membrane mold. This allowed them to achieve a high packing density of 50 vol% without compromising the flexibility of the membrane. They also used a fine-pitch magnetization pattern to further reduce the self-demagnetization effect and improve the actuator performance. The experimental results show that the fabricated EMMA (12 mm × 12 mm × 1.1 mm) can generate a maximum force of 2.2 mN and a maximum displacement of 100 μm at a power consumption of 4 W. In Figure 4b, Jia et al. present a MEMS electromagnetic swing-type actuator for optical switches, which has a compact size of 5.1 × 5.1 × 5.3 mm^3^ and consists of two stators, a swing disc (rotator), a rotating shaft, and protective covers. The actuator uses multi-winding stators and a multipole rotator to increase the output torque. The working principle and magnetic circuit of the actuator show that the magnetic flux density in the air gap between the stators and swing disc is crucial for the output torque. NiFe alloy magnetic cores are embedded in the windings to increase the magnetic flux density. The fabricated actuator prototype demonstrates a large output torque (40 μNm), fast response (5 ms), and a large swing angle (22°), making it suitable for optical switch applications [34]. Zhi et al. report a micro-electromagnetic linear actuator consisting of a stator with two-phase microcoils, a slider with thick NdFeB permanent magnets, a guide layer, and tunneling magneto-resistance (TMR) sensors for position monitoring. Actuation is achieved through the Lorentz force between the microcoils and the permanent magnets on the slider. The stator and guide layer are fabricated using MEMS processes as show in Figure 4c. The actuator is capable of achieving a slider speed of 60 mm/s with a driving frequency up to 500 Hz. The position monitoring using TMR sensors achieves a maximum error of 2.2% for a 4.5 mm stroke [35].

### 2.3. Typical Design Configurations and Geometries

The design configuration and geometric arrangement of MEMS electromagnetic actuators fundamentally determine their operational characteristics, including the actuation force, displacement range, frequency response, and overall efficiency. Recent advancements in microfabrication technology have facilitated diverse and increasingly complex geometrical configurations, each tailored to specific performance requirements and application scenarios. This section elaborates on typical actuator design configurations and geometric considerations that significantly impact actuator functionality, providing insights into their practical implementation in microsystem applications.

Planar coil geometries represent one of the most prevalent configurations utilized in MEMS electromagnetic actuators, primarily due to their compatibility with standard microfabrication processes and straightforward integration onto silicon-based substrates [36,37]. These configurations usually incorporate spiral-shaped planar coils patterned by lithography and electroplating techniques, enabling precise control over the coil dimensions, turn density, and inter-wire spacing. Planar coil actuators typically exhibit favorable force density and ease of fabrication, making them particularly suited for applications such as micromirrors, microswitches, and micro-relays [38,39]. However, planar configurations often present limitations in terms of magnetic field strength due to spatial constraints, necessitating the optimization of coil dimensions and current-carrying capabilities to achieve the desired performance [40].

**Figure 5:**
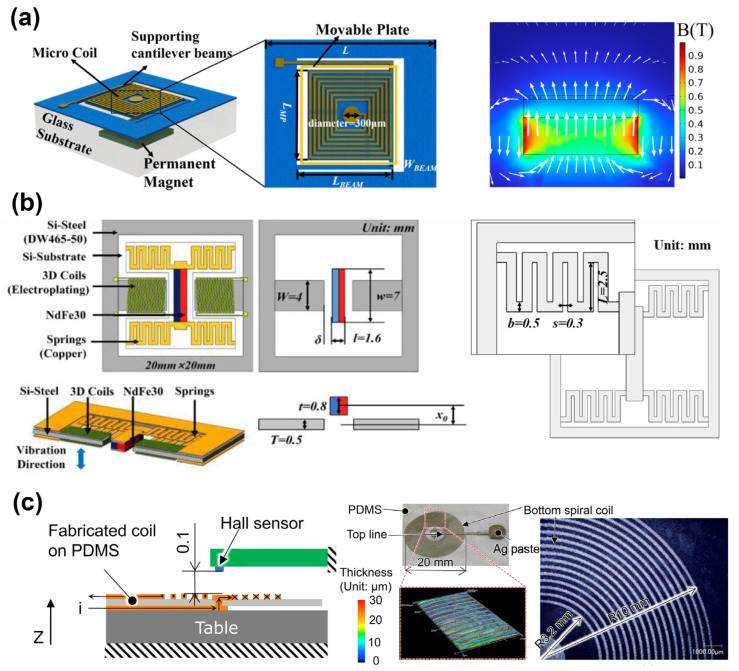
(**a**) Design of the electromagnetic actuator (EMA) and the magnetic flux density generated by a coil superimposed on a permanent magnet [41]. (**b**) Overall structure of the micro VEH and spring geometric parameters [42]. (**c**) Vertical magnetic flux density generated by the current-carrying coil and photograph of the coil and close-up of a spiral coil [43]. Reprinted/adapted with permission from Ref. [43]. 2025, Elsevier.

Zhou et al. present a flexible electromagnetic actuator for tunable terahertz metamaterials consisting of supporting cantilever beams, an integrated coil positioned above the cantilever’s movable plate, and a permanent magnet located beneath the plate to generate a static magnetic field as shown in Figure 5a [41]. By utilizing flexible polyimide as the substrate material, the fabrication process is simplified compared to traditional silicon-based micromachining techniques. The EMA can achieve a displacement of up to 250 μm under a 100 mA current, enabling its integration with metamaterial structures to form compact, tunable terahertz absorbers. The large displacement, rapid response, and immunity to nonlinear effects make this EMA a promising candidate for various MEMS applications, including reconfigurable metamaterials. Tao et al. develop a micro-electromagnetic vibration energy collector based on 3D MEMS coil and silicon steel sheet core structure, which effectively improves the energy collection efficiency and output power as shown in Figure 5b [42]. The effects of the air gap and initial magnet position offset on the dynamic characteristics and output performance of the system are analyzed by numerical simulation, and it is found that there is an optimal initial position offset to maximize the output power. The proposed theoretical model and design method can be used to guide the design and optimization of devices with similar structures and provide a new idea and technical route for MEMS electromagnetic vibration energy-harvesting technology. Qi et al. develop a flexible coil based on a conductive polymer composite (CPC) for PDMS-based soft electromagnetic microactuators (SEMMAs), which gained attention for portable microfluidic systems due to their low drive voltage and high response, but membranes with rigid permanent magnets or conductive liquids have impaired flexibility [43]. They propose using a screen-printing technique to print a CPC-based coil directly on a PDMS membrane, which can maintain the flexibility of the moving part. The printed CPC coil has a small thickness and low Young’s modulus, ensuring it does not significantly affect the membrane’s flexibility. As shown in Figure 5c, a prototype SEMMA with a 19.5 μm thick spiral CPC-based coil on a Φ30 mm × 0.1 mm PDMS substrate is fabricated and evaluated, demonstrating static displacements and asymmetrical displacements under different voltage inputs.

Solenoidal coil geometries constitute another widely explored configuration, distinguished by their three-dimensional coil winding arrangement, which provides enhanced magnetic field density and greater force generation capability compared to planar configurations [28,44,45]. The solenoidal design involves fabricating coils around cylindrical or similarly shaped magnetic cores, often realized through advanced fabrication techniques such as LIGA, deep reactive ion etching (DRIE), and additive manufacturing. This geometry yields an increased actuation force, improved magnetic flux concentration, and superior displacement capabilities [46,47]. Consequently, solenoidal actuators are widely employed in applications demanding robust actuation, such as microvalves, micropumps, and precision positioning devices. Nonetheless, the complexity of three-dimensional coil fabrication poses significant challenges, including difficulties associated with uniform coil winding, alignment accuracy, and process repeatability [48].

**Figure 6:**
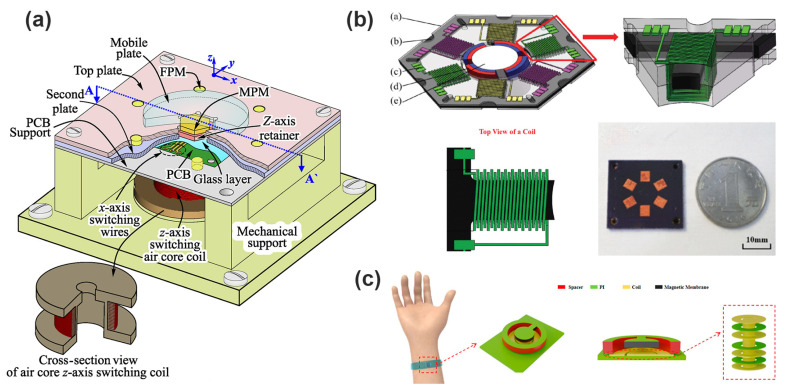
(**a**) Representation of the micro-positioning device. [28]. (**b**) Structure of the micromotor and details of the stator slot and top view of a coil and magnetic flux density distribution [45]. (**c**) Principle view, sectional view, and magnetic coil of electromagnetically actuated devices [44]. Reprinted/adapted with permission from Ref. [44]. Copyright 2025 American Chemical Society.

Deshmukh et al. presents an innovative planar micro-positioning device leveraging a 3D digital electromagnetic actuator, featuring twelve discrete positions across two vertical levels through a hexagonal mobile magnet design as shown in Figure 6a. By introducing both stick-slip and lift-mode actuation mechanisms, the device achieves versatile and precise xy-plane positioning, making it highly suitable for micro-manipulation applications. The integration of discrete digital actuation with 3D mobility enables open-loop control without the need for position feedback sensors, thereby simplifying the control scheme while maintaining high positioning repeatability. This work exemplifies the potential of digital electromagnetic actuators in miniaturized systems requiring reliable and flexible micro-positioning [28]. Tao et al. present the design, fabrication, and characterization of a radial-flux permanent magnet (PM) micromotor with 3D solenoid iron-core MEMS in-chip coils shown in Figure 6b. The micromotor has a conventional large-scale brushless DC motor structure, with a four-pole rotor and a six-slot stator. The key innovation is the use of high aspect ratio 3D solenoid coils with iron cores, which provide higher inductance and lower magnetic resistivity compared to planar spiral coils or air-core solenoid coils. The micromotor exhibits high power density and efficiency due to the excellent heat dissipation, low magnetic flux leakage, and high current-carrying capacity of the iron-core coils [45]. Dong et al. propose a flexible electromagnetic actuator based on the combination of NdFeB magnetic particles and PDMS polymer shown in Figure 6c [44]. Through the three-dimensional microcoil structure, vibration and non-vibration dual-mode operation is realized. The innovative combination of flexible materials and high-performance magnetic particles enables highly sensitive displacement feedback, with a maximum displacement of 651 μm in AC vibration mode and 187 μm in DC non-vibration mode. The actuator not only has excellent flexibility and comfort but also shows fast response, high sensitivity, and easy dual-mode switching, which is suitable for haptic feedback devices, virtual reality systems, and wearable medical devices.

Another notable actuator geometry involves magnetic cantilever beam configurations, where a thin cantilever structure, typically fabricated from soft magnetic materials, serves as the movable component actuated by electromagnetic forces. This configuration leverages magnetic attraction or repulsion phenomena, with the cantilever deflecting under the influence of the magnetic forces generated by the integrated coils or external magnetic fields [49,50]. Cantilever geometries are advantageous due to their straightforward design, low operating voltages, high sensitivity, and rapid response times. Such actuators have proven particularly beneficial in optical switching, sensor applications, and biomedical devices requiring precise and controllable microscale displacements. Nevertheless, challenges such as residual stress-induced deformation, limited stroke length, and susceptibility to external disturbances must be carefully addressed during the design and fabrication phases [51,52,53].

**Figure 7:**
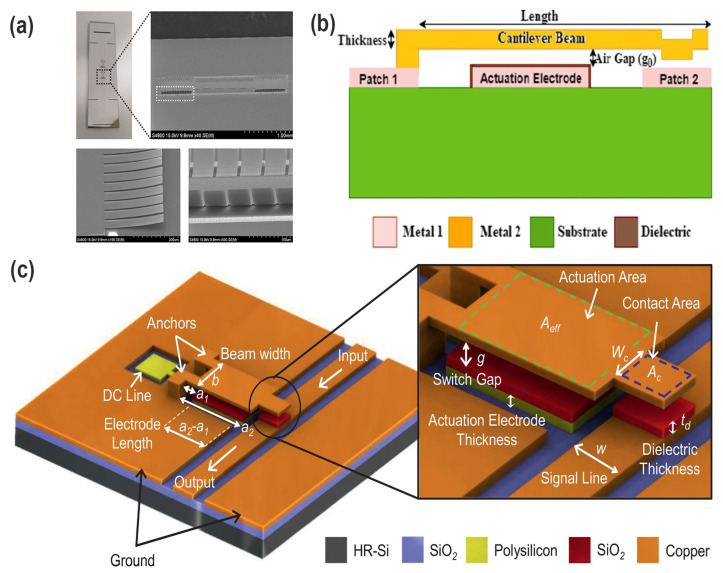
(**a**) Photograph of a fabricated MEMS frequency-tunable absorber sample [54]. (**b**) Schematic representation of the cantilever beam type series ohmic DC RF MEMS switch [49]. (**c**) The 3D modeling of the proposed cantilever-type shunt RF-MEMS switch [50].

Chung et al. present a MEMS-driven millimeter-wave metamaterial absorber featuring tunable resonance characteristics through electrostatically actuated cantilever arrays shown in Figure 7a. By integrating stress-engineered MEMS cantilevers into split-ring resonator (SRR) unit cells, the absorber achieves significant frequency tunability from 28 GHz to 25.5 GHz with a low actuation voltage of 15 V. The work demonstrates a highly effective solution for dynamic electromagnetic control in compact, CMOS-compatible platforms, offering new opportunities for reconfigurable RF and stealth applications [54].

Kasambe et al. propose a single-step cantilever beam design based on a “single-step broadening” structure to reduce the pull-in voltage of a MEMS RF switch, significantly optimizing its mechanical properties compared to conventional rectangular beam structures, as shown in Figure 7b. The Conjugate Beam Method and Rayleigh method were used to establish a theoretical model of the key parameters. The final obtained single girder structure can reduce the pull-in voltage to 11.20 V (corresponding stiffness 2.18 N/m), which is significantly improved compared with the traditional rectangular beam structure (14.16 V, stiffness 3.36 N/m), and the resonance frequency is 15.84 kHz, while the stress is 14.8 MPa. These parameters meet the requirements of device stability and reliability [49].

Saleh et al. propose the design and optimization of a cantilever-type RF-MEMS shunt switch tailored for 5G applications. By employing a fixed-free cantilever structure rather than conventional membrane-based designs, the switch achieves significantly reduced actuation voltages (as low as 9.1 V) while maintaining excellent RF performance (Figure 7c). Finite element simulations in COMSOL and HFSS were used to simultaneously optimize electromechanical and RF characteristics. The proposed switch demonstrates a low insertion loss of −0.065 dB and high isolation of −44.7 dB at 30 GHz. Furthermore, a systematic analysis of cantilever geometry, switching time, mechanical stress, and reliability ensures robust device operation. This work provides a compelling solution for integrating low-voltage, high-performance RF-MEMS switches into next-generation millimeter-wave systems, offering valuable design strategies and modeling insights for MEMS researchers [50]. 

Additionally, gap-closing actuators represent a specific geometry frequently employed within MEMS electromagnetic actuators, characterized by their operation based on the magnetic attraction or repulsion forces across narrow gaps between fixed and movable structures. By adjusting the gap width, magnetic field distribution, and geometry of electrodes and cores, gap-closing configurations provide substantial controllability of the force-displacement characteristics, ideal for applications requiring precise and adjustable micropositioning. Applications of gap-closing actuator geometries include microgrippers, tunable micro-optical components, and advanced microfluidic control devices. Geometric optimization of MEMS electromagnetic actuators is frequently supported by computational modeling and finite element analysis (FEA), enabling detailed assessment and refinement of actuator structures prior to fabrication. Numerical simulations allow designers to predict magnetic field distributions, force outputs, thermal behavior, and structural stresses under realistic operational conditions, significantly reducing the design iteration time and enhancing actuator reliability. Coupling advanced simulation tools with iterative experimental validation fosters the development of highly optimized geometries tailored explicitly for targeted application needs [55,56,57].

## 3. Fabrication Techniques and Technological Advances

The performance, functionality, and integration capabilities of MEMS electromagnetic actuators are strongly dependent on their fabrication techniques, material selection, and associated manufacturing processes. MEMS fabrication relied predominantly on standard semiconductor micromachining processes such as photolithography, etching, and thin-film deposition. However, as MEMS applications have diversified into more demanding domains, conventional fabrication methods have faced challenges regarding process complexity, scalability, dimensional precision, and material compatibility, necessitating significant technological advancements. Recently, novel microfabrication strategies and improved methodologies have emerged, allowing for more intricate geometries, enhanced structural precision, and superior material properties that collectively contribute to actuator performance improvement. Techniques such as DRIE, electroplating, and lithographie galvanoformung abformung (LIGA), alongside additive manufacturing, have introduced new degrees of design freedom, cost efficiency, and production scalability [58,59,60]. Additionally, recent developments in materials science, particularly the synthesis and deposition of advanced soft magnetic alloys and magnetic nanocomposites, have played pivotal roles in elevating the magnetic and mechanical properties critical for actuator efficiency [61,62].

### 3.1. MEMS Fabrication Methods Relevant to Electromagnetic Actuators

Traditional micromachining techniques primarily include bulk micromachining and surface micromachining. Bulk micromachining utilizes the selective removal of substrate materials—commonly silicon—to create microstructures via processes such as wet chemical etching and DRIE. DRIE has become essential for fabricating vertical structures and complex geometries required in electromagnetic actuators, owing to its ability to achieve high aspect ratios, vertical sidewalls, and precise dimensional control. Surface micromachining builds structures layer-by-layer through the deposition and patterning of thin films. Thin-film deposition methods such as physical vapor deposition, chemical vapor deposition, sputtering, and electroplating enable the formation of conductive coils and magnetic materials crucial to actuator functionality [63,64,65].

**Figure 8:**
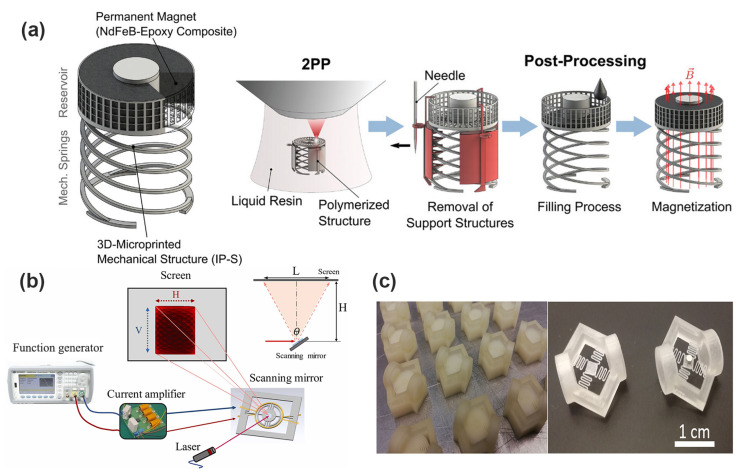
(**a**) Illustration of the 3D-printed microactuator consisting of a mechanical spring attached to a permanent magnet and the fabrication process involves the 3D-printing of the mechanical structure via 2PP, as well as three post-processing steps [69]. (**b**) Diagram of the test system of a scanning mirror [70]. Reprinted/adapted with permission from Ref. [70]. 2025, Elsevier. (**c**) Vibrational 3D-printed MEMS-type energy harvesters [74].

More recently, advanced micromachining techniques have emerged that can overcome the limitations associated with conventional methods. The LIGA process—comprising lithography, electroforming, and molding—has gained widespread adoption due to its capability to produce microstructures with exceptionally high aspect ratios and superior structural accuracy. Through LIGA, actuator components such as coils and magnetic cores with intricate geometries and improved mechanical robustness can be reliably fabricated. Additionally, wafer-level bonding techniques, including anodic bonding and eutectic bonding, facilitate the integration of heterogeneous materials (such as magnetic alloys and polymers) onto silicon substrates, significantly broadening material selection and enhancing actuator performance [66,67,68]. Rothermel et al. propose a new type of 3D printed magnetically driven micro-actuator with a diameter of 500 µm. As shown in Figure 8a, this micro-actuator structure adopts a mechanical spring design, which can achieve large displacement continuous positioning. The driving experiment shows that the micro-actuator can produce a displacement response of 69.1 to 88.9 μm, but there is a hysteresis behavior, which is attributed to the viscoelastic and magnetic properties of the material. By real-time demagnetization and closed-loop control, the hysteresis behavior was solved, and high repeatability and precise positioning were achieved [69]. As shown in Figure 8b, Li et al. innovatively propose an electromagnetic dual-axis scanning mirror based on laser patterning technology, which breaks through the limitations of high cost, complex process, and long cycle in traditional MEMS micromirror manufacturing. The mirror body structure is printed by ABS plastic FDM, and the driving coil is made by a UV laser directly patterning copper foil, realizing high efficiency, low cost, and fast manufacturing. The scanning mirror structure contains two sets of independent drive coils, which are used to control the internal and external torsion beam, respectively, to achieve a true dual-axis independent drive, avoiding the electromagnetic crosstalk caused by the traditional single-coil structure and effectively improving the freedom of motion and control accuracy. The device under resonant excitation (119 Hz and 250 Hz) can achieve a 32.4° vertical scanning angle and 16.5° horizontal scanning angle, with a large aperture (maximum 10 mm), good scanning range, and superior performance in similar electromagnetic scanning mirrors [70].

Additive manufacturing methods, also known as three-dimensional (3D) printing, represent another rapidly growing area in MEMS fabrication. Techniques such as stereolithography (SLA), two-photon polymerization (TPP), selective laser melting (SLM), and direct ink writing (DIW) have recently demonstrated substantial potential in fabricating MEMS electromagnetic actuators. These techniques offer substantial advantages, including simplified processing steps, rapid prototyping capabilities, customization potential, and reduced fabrication costs. In particular, additive manufacturing enables complex 3D geometries and integrated architectures that are difficult or impossible to achieve through traditional microfabrication, thus significantly enhancing actuator functionality and miniaturization [71,72,73].

Kawa et al. used inkjet 3D printing technology to build a micro-electromagnetic energy collector with a structural size of less than 1 cm^3^, which, for the first time, combined the elastic microstructure with commercial microcoils and neodymium magnets to form a vibration-driven energy-harvesting system shown in Figure 8c. The design includes microsprings with different geometric shapes (200 µm and 400 µm thickness) and a central proton structure. The resonance frequency is predicted by numerical simulation, combined with the actual elastic modulus test, which greatly improves the accuracy of structural modeling. The measured resonance frequency is between 95 and 340 Hz, and the bandwidth is up to 23 Hz. Under 1 g vibration conditions and optimal resistance matching, the maximum output power of 23.7 µW is obtained, and the corresponding power density is close to 600 µW/cm^3^, which is at the forefront of the current performance of the micro-electromagnetic energy collector [74].

Material selection plays an equally vital role in the fabrication of MEMS electromagnetic actuators. Soft magnetic materials—such as permalloy, cobalt-based alloys, and amorphous metals—are extensively utilized due to their high permeability, low coercivity, and minimal hysteresis losses. The deposition of these magnetic materials typically involves sputtering, electroplating, or electroless plating methods, ensuring compatibility with existing MEMS fabrication processes. In addition, conductive materials, notably copper and aluminum, are commonly used for coil fabrication to achieve optimal electrical conductivity and reduced resistive losses [75,76].

### 3.2. Smart and Adaptive Actuator Systems

The ongoing miniaturization and complexity of MEMS have accelerated the integration of smart and adaptive capabilities into MEMS electromagnetic actuators, significantly expanding their functional versatility and application scope. Smart actuator systems are characterized by their ability to sense, analyze, and autonomously adjust their operational parameters in response to environmental changes or internal feedback, thereby ensuring optimized performance, increased reliability, and greater energy efficiency. Recent research efforts have concentrated on embedding sensing mechanisms, integrating closed-loop control algorithms, and developing adaptive materials and structures to facilitate actuator responsiveness and intelligence [77,78].

Embedding microscale sensing components within actuator structures has become a prominent strategy for enabling real-time monitoring and feedback control. Integrated sensors such as strain gauges, capacitive position sensors, or piezoresistive elements can detect key operational parameters, including displacement, force, temperature, and structural deformation. These sensors provide critical data streams to inform the actuator control system, enabling precise adjustments to electromagnetic driving signals. Consequently, actuator systems can dynamically respond to varying load conditions, external disturbances, or internal degradation, effectively enhancing operational stability and lifespan [79,80,81].

The implementation of closed-loop control methodologies represents another key advancement in achieving actuator adaptability. Through integrated control electronics and advanced control algorithms—including proportional-integral-derivative (PID) control, adaptive control, and model predictive control—MEMS electromagnetic actuators gain the capability for real-time, automated adjustments to maintain desired output characteristics [82,83]. Adaptive control algorithms, in particular, enable actuators to learn and respond proactively to changing environmental conditions or actuator wear, ensuring consistent system performance despite uncertainty or variability. Recent progress in embedded microcontroller technology and low-power integrated circuits has further facilitated the practical implementation of these sophisticated control strategies at the MEMS scale [84,85].

The incorporation of adaptive and multifunctional materials also significantly enhances the intelligence of MEMS electromagnetic actuator systems. Smart materials—such as shape memory alloys (SMAs), magnetostrictive materials, electroactive polymers (EAPs), and piezoelectric composites—can actively alter their mechanical and electromagnetic properties in response to external stimuli, contributing additional adaptive functionality beyond conventional actuator capabilities. Integrating these materials within electromagnetic actuator configurations has yielded actuator systems with embedded self-healing, shape-adaptive, or energy-harvesting capabilities. Such adaptive behaviors enable actuators to autonomously respond to mechanical strain, temperature fluctuations, or electromagnetic interference, providing enhanced robustness and multifunctionality [86,87].

**Figure 9:**
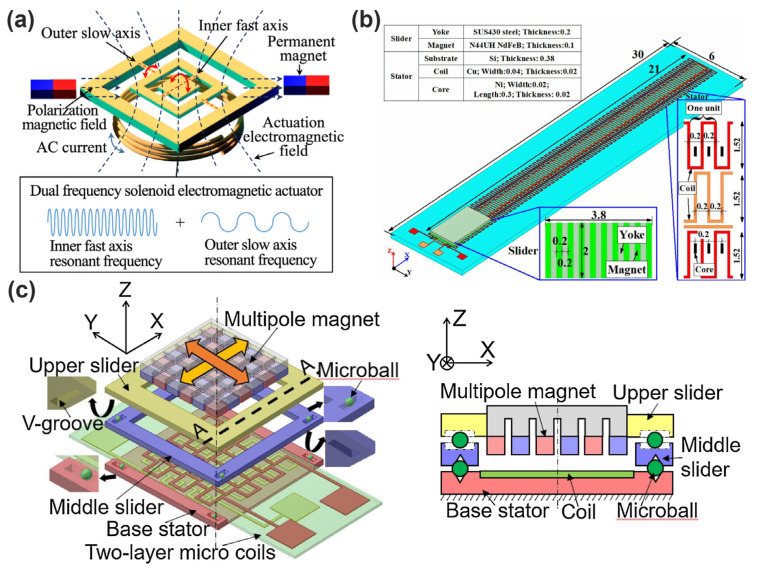
(**a**) Schematic of the proposed 2D Fe-based MG micromirror driven by an electromagnetic actuator [90]. Reprinted/adapted with permission from Ref. [90]. 2025, IOP Publishing on behalf of the Japan Society of Applied Physics. (**b**) Microactuator overall design and feature size (in mm unit) [78]. Reprinted/adapted with permission from Ref. [78]. 2025, IOP Publishing Ltd. (**c**) Schematic of the proposed 2-DOF micro-electromagnetic actuator [91]. Reprinted/adapted with permission from Ref. [91]. 2025, Elsevier.

Furthermore, recent advancements in wireless communication and Internet of Things (IoT) integration have accelerated the development of networked MEMS electromagnetic actuators, which can communicate performance data, receive external commands, and cooperate within actuator arrays. Such distributed and coordinated actuator networks facilitate advanced cooperative behaviors, enabling adaptive shape control, distributed force generation, or synchronized movements that are beneficial in applications ranging from microrobotics and biomedical implants to smart microfluidic systems and adaptable optical devices [88,89]. Ou et al. propose a 2D MEMS electromagnetic micromirror based on Fe-based amorphous metal (MG) shown in Figure 9a. Fe-based MG materials have high strength, low Young’s modulus, and high permeability and are used not only as torsional beam structures of micromirrors but also as magnetizable driving films, which solve the limitations of traditional silicon-based materials in reliability and driving efficiency. This research has the potential to embed MEMS micromirrors into lightweight, high-precision, low-power IoT smart terminals, especially suitable for edge intelligent devices with strict requirements for volume, power consumption, and reliability [90]. Zhi et al. propose a micro-linear electromagnetic actuator with an innovative structure, which realizes self-alignment and self-attachment between the slider and the stator through the attraction between the permanent magnet (NdFeB) and the ferromagnetic core (Ni) shown in Figure 9b. The assembly convenience and stability of the device are effectively improved. The actuator combines the bidirectional Lorentz force between the magnet and the coil with the unidirectional attraction between the magnet and the core, making the drive more efficient while providing a stable attachment capability that can withstand 30 G centrifugal acceleration without power [78]. Han et al. present a novel MEMS electromagnetic microactuator with large-stroke, low-voltage, and precise 2-DOF (degrees of freedom) motion capability as shown in Figure 9c. Its large-stroke, in-plane movement with micro-Newton-level control precision is ideal for microrobotics and smart sensors in IoT systems that demand fine mechanical control in wearables, implantables, and autonomous inspection tools [91].

## 4. Key Applications

Advancements in MEMS electromagnetic actuators have catalyzed transformative developments across a wide spectrum of microscale technologies, enabling applications previously limited by conventional actuator approaches. Leveraging distinctive advantages such as high force density, precise controllability, fast response, and low-voltage operation, MEMS electromagnetic actuators have found increasing utility in diverse high-impact application domains. In micro-optical systems and micromirror technologies, MEMS electromagnetic actuators play an indispensable role, offering rapid, precise, and repeatable positioning capabilities crucial for optical signal steering and adaptive optics. Recent actuator advancements have significantly enhanced the speed and angular accuracy of MEMS-based micromirrors, enabling sophisticated optical devices and systems, including optical switches, adaptive optics, and optical coherence tomography (OCT).

The field of microrobotics and precision micromanipulation has similarly benefited from advances in electromagnetic actuator technology. The integration of high-performance actuators into microrobotic systems has enabled precise manipulation and controlled motion at microscopic scales, essential for applications ranging from minimally invasive surgery and biomedical sample handling to automated microassembly and cell manipulation tasks. Recent developments in actuator miniaturization and control algorithms have further extended the functional capabilities of microrobotic systems, supporting complex, high-precision operations that were previously unattainable.

Finally, MEMS electromagnetic actuators have emerged as critical components within microfluidic devices, where precise control over fluid movement, valve operation, and pump functions is paramount. Improved actuator performance in terms of responsiveness and force output has facilitated advancements in lab-on-a-chip devices, biomedical diagnostics, and point-of-care testing platforms. The ability to reliably manipulate fluids at the microscale continues to broaden the scope of microfluidic applications, underscoring the significant role that electromagnetic actuator innovation plays in shaping future microfluidic technologies.

### 4.1. Micro-Optical Systems and Micromirrors

MEMS micromirrors actuated via electromagnetic principles provide precise angular positioning, rapid dynamic response, and high mechanical reliability, which are attributes that are critical for sophisticated optical applications. Electromagnetically actuated micromirrors operate primarily through Lorentz force, magnetic attraction/repulsion, or inductive interactions, offering unique advantages in speed, accuracy, and actuation range compared to electrostatic or piezoelectric counterparts. Specifically, electromagnetic actuation methods enable large deflection angles and superior mechanical robustness due to the relatively high driving forces achievable at moderate voltages and currents. Such advantages have driven the incorporation of MEMS electromagnetic micromirrors in diverse fields including optical communication systems, adaptive optics, OCT, laser scanning displays, and optical switches [92,93].

In optical communication applications, MEMS electromagnetic micromirrors serve as critical components for optical switching, beam steering, and signal routing in fiber-optic networks. Recent developments in compact design, integration with sophisticated feedback control systems, and improved magnetic materials have led to significant improvements in the switching speed, repeatability, and long-term stability. This facilitates higher data transmission capacities, enhances network reliability, and reduces latency in optical communication systems. Adaptive optics systems also significantly benefit from MEMS electromagnetic micromirror technologies. The ability to precisely and dynamically control the mirror position enables real-time wavefront correction, essential for applications including astronomy, biomedical imaging, and advanced microscopy. Advances in actuator control algorithms and integration strategies have further improved the response time, positioning accuracy, and overall system robustness, enhancing image clarity and resolution beyond the capabilities of traditional optical methods [94,95].

Moreover, MEMS electromagnetic micromirrors are integral in biomedical optical imaging techniques such as OCT, endoscopic imaging, and confocal microscopy. The precise actuation and rapid scanning capabilities afforded by these micromirrors improve image acquisition rates, scanning uniformity, and image quality. Recent efforts have focused on optimizing actuator geometry, material selection, and fabrication techniques to achieve higher resonance frequencies and reduced power consumption, making MEMS electromagnetic micromirrors increasingly practical for portable and clinical imaging systems [96,97].

**Figure 10:**
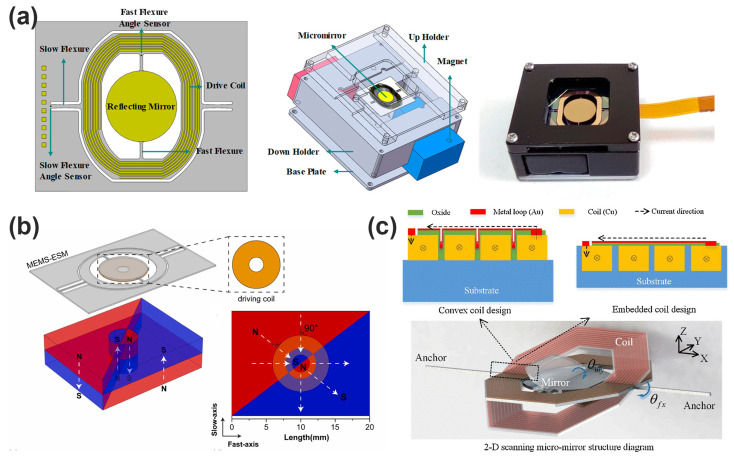
(**a**) The electromagnetic MEMS micromirror and package [98]. (**b**) The proposed PMS system and top view of the assembled coil and magnet and the direction of the magnetic field lines [99]. (**c**) Diagram of convex coil design, embedded coil design and two-dimensional electromagnetic driving scanning micromirror [92]. Reprinted/adapted with permission from Ref. [92]. 2025, Elsevier.

Qian et al. present a significant advancement in enhancing the vibration robustness of electromagnetic MEMS micromirrors, which are essential components in automotive LiDAR systems shown in Figure 10a. They implement a mechanical low-pass filter (LPF) using a beryllium–copper alloy to attenuate detrimental resonances—particularly the high-stress piston mode around 1057 Hz. The study demonstrates that the LPF effectively reduces mechanical stress by approximately 49% during high-frequency Z-directional vibrations, thereby enhancing micromirror reliability. The proposed LPF offers a practical and low-cost solution for improving the operational resilience of large-aperture electromagnetic MEMS mirrors in harsh automotive environments [98]. As shown in Figure 10b, Liu et al. propose a permanent magnet structure based on the principle of flux concentration, which significantly improves the magnetic induction intensity. Under the same package volume, the structure can reduce the total driving current by 31.3% and 55.0% compared with the traditional L and I magnets, respectively. Through the optimization design of the cutting angle and the comparative analysis of the experimental system, the final measured power consumption is only 27.68 mW, which is a very advantageous low power scheme in the current dual-axis MEMS mirror. Its drive currents (83.32 mA and 65.99 mA) are also well below industry standards, significantly improving long-term system safety [99]. Jiang et al. present a design and fabrication of a 2D micromirror with large electromagnetic driving forces shown in Figure 10c. Adopting an embedded coil design, which can provide a larger electromagnetic driving force compared to the protruding coil design. Adopting a local deposition and lift-off process to partially alleviate the stress problem caused by the difference in thermal expansion coefficients. The fabricated 3mm micromirror can achieve a horizontal swing angle of ±23.6° and a vertical swing angle of ±5.7° under a 5 V voltage [92].

Despite considerable progress, challenges remain in terms of minimizing power requirements, reducing actuator footprint, and improving long-term reliability. Research continues to explore innovative materials, improved fabrication methods, and advanced control strategies to further enhance actuator performance and applicability. The ongoing development of MEMS electromagnetic micromirror technologies thus promises substantial future advancements in micro-optical systems, significantly influencing both academic research and commercial applications.

### 4.2. Microrobotics and Precision Micromanipulation

Microrobotics represents an increasingly prominent field that relies significantly on precise, reliable, and miniaturized actuator technologies. MEMS-based electromagnetic actuators have emerged as vital components enabling microrobotic systems and precision micromanipulation tasks, owing to their advantages in force density, scalability, rapid response, and integration capability. These features are particularly critical when considering robotic systems operating at the microscale, which require high accuracy, precise positioning, robust force feedback, and efficient energy utilization in confined environments [100,101].

**Figure 11:**
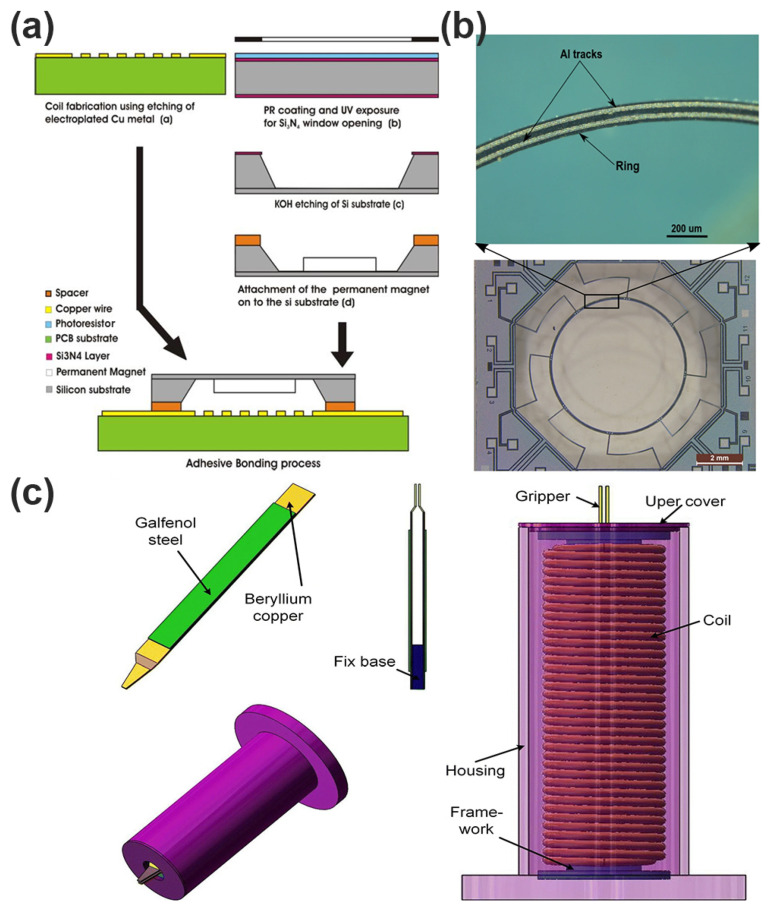
(**a**) The process flow for the fabrication of electromagnetic actuator [100]. (**b**) Microfabricated ring resonator with functional metal tracks formed over the suspended structure, and a closer view of the electrode pattern on the ring structure [101]. (**c**) Structure diagram of the micro-gripper including Galfenol composites cantilever beam, two arms of the micro-gripper, 3-D model of the magnetostrictive micro-gripper and each part of the magnetostrictive micro-gripper [107].

Electromagnetic actuation has facilitated the realization of advanced microrobotic platforms capable of performing complex tasks such as microassembly, biomedical interventions, and microscale manufacturing. Specifically, microrobotic grippers and manipulators that leverage electromagnetic actuation have demonstrated significant improvements in grip force, manipulation precision, and operational stability compared to electrostatic or thermal actuators. One key advantage of MEMS electromagnetic actuators in precision micromanipulation is their ability to provide well-defined force control, which is essential for delicate tasks such as cell manipulation, microinjection, or the microassembly of delicate MEMS components [102,103]. Recent research has highlighted novel actuator configurations, including multi-degree-of-freedom systems, compliant electromagnetic actuators, and integrated actuator–sensor platforms. For example, compliant electromagnetic actuators employing flexure-based mechanisms allow for controlled, smooth, and backlash-free motion essential for precision positioning tasks. Additionally, the integration of sensor feedback loops—such as capacitive, optical, or magnetic position sensing—has enabled closed-loop control strategies, enhancing the accuracy and repeatability of micromanipulation operations [104,105,106]. Pawinanto et al. present an electromagnetic microactuator with a silicon membrane for use in a fluid pump system for drug delivery applications shown in Figure 11a. The actuator consists of an electromagnetic coil and a magneto-mechanical part with a thin silicon membrane and a permanent magnet. The silicon membrane is deformed by the magnetic force generated by the electromagnetic coil, enabling fluid pumping. The 20 μm thin silicon membrane can achieve a maximum deflection of 4.5 μm with a power consumption of around 0.8 W, which is suitable for a reliable fluid pump in a continuous drug delivery system [100].

Another significant development in MEMS electromagnetic actuators for microrobotics is their capability for untethered or minimally tethered robotic platforms, especially in biomedical and in vivo applications. Magnetic attraction and repulsion actuators have been employed in the development of magnetically guided or remotely controlled microrobots, capable of precise navigation and manipulation within constrained biological environments. These actuators offer the distinct advantage of non-contact actuation and control, minimizing external mechanical interference and enabling novel functionalities such as targeted drug delivery, microsurgery, and minimally invasive diagnostic procedures. Reddy et al. propose a method for manufacturing MEMS ring resonators with high current carrying capacity from a single silicon-on-insulator (SOI) wafer shown in Figure 11b. The reported method describes a novel technique to increase the current handling capability of the metal tracks by introducing a conformal electromigration suppression layer. The experimental results show that the current carrying capacity of the metal lines is improved by at least 300%. The proposed technique is implemented in realizing an electromagnetic induction-based ring gyroscope, and the electrical and mechanical responses of the ring resonator are presented [101].

Nevertheless, the adoption of MEMS electromagnetic actuators within microrobotics continues to face several challenges. Key among these are achieving sufficient miniaturization and integration to facilitate highly compact systems, optimizing energy consumption and dissipation to support autonomous operation, and developing sophisticated control strategies for reliable and precise manipulation under dynamic conditions. Addressing these challenges through continued advancements in actuator design, materials science, control systems, and microfabrication processes is essential to unlocking the full potential of MEMS electromagnetic actuators in future microrobotic and precision micromanipulation applications. Zhao et al. propose a new type of magnetostrictive micro-gripper using an iron-gallium alloy (Galfenol) composite cantilever beam as the actuator, with a simple structure and fast response, as shown in Figure 11c. This micro-gripper can achieve a maximum gripping gap of 250μm using a drive current of up to 1A, with a large gripping range and high resolution. The authors simulated the magnetic field distribution and claw displacement of the micro-gripper, and established a revised theoretical model to accurately predict the claw displacement. A multi-stage driving method is proposed, which can effectively eliminate vibration and improve the stability and grasping performance of the micro-gripper. This micro-gripper is suitable for micro-operation and micro-assembly applications, with advantages such as simple structure and fast response [107].

### 4.3. Microfluidic Components

Microfluidics, characterized by the precise manipulation of minute volumes of fluids within microscale channels, has become a cornerstone of numerous biomedical, chemical, and analytical applications. Central to effective microfluidic system performance is the capability for precise fluid actuation, accurate flow control, and efficient mixing. MEMS-based electromagnetic actuators have increasingly been adopted within microfluidic systems due to their ability to deliver substantial actuation forces, rapid response times, and reliable performance at microscale dimensions, thus addressing the critical requirements of microfluidic platforms [108,109].

MEMS electromagnetic actuators integrated into microfluidic devices primarily serve functions such as micropumping, microvalving, droplet manipulation, and fluid mixing enhancement. For example, electromagnetic micropumps exploit magnetic fields and coil-induced electromagnetic forces to drive fluids within microchannels [110,111]. Compared to conventional micropumps based on electrostatic or piezoelectric mechanisms, electromagnetic micropumps demonstrate superior performance characteristics, including larger stroke volumes, higher achievable pressures, and robustness against particle contamination. Furthermore, electromagnetic micropumps often operate effectively at lower voltages, simplifying integration with portable or battery-powered microfluidic devices [112,113,114].

**Figure 12:**
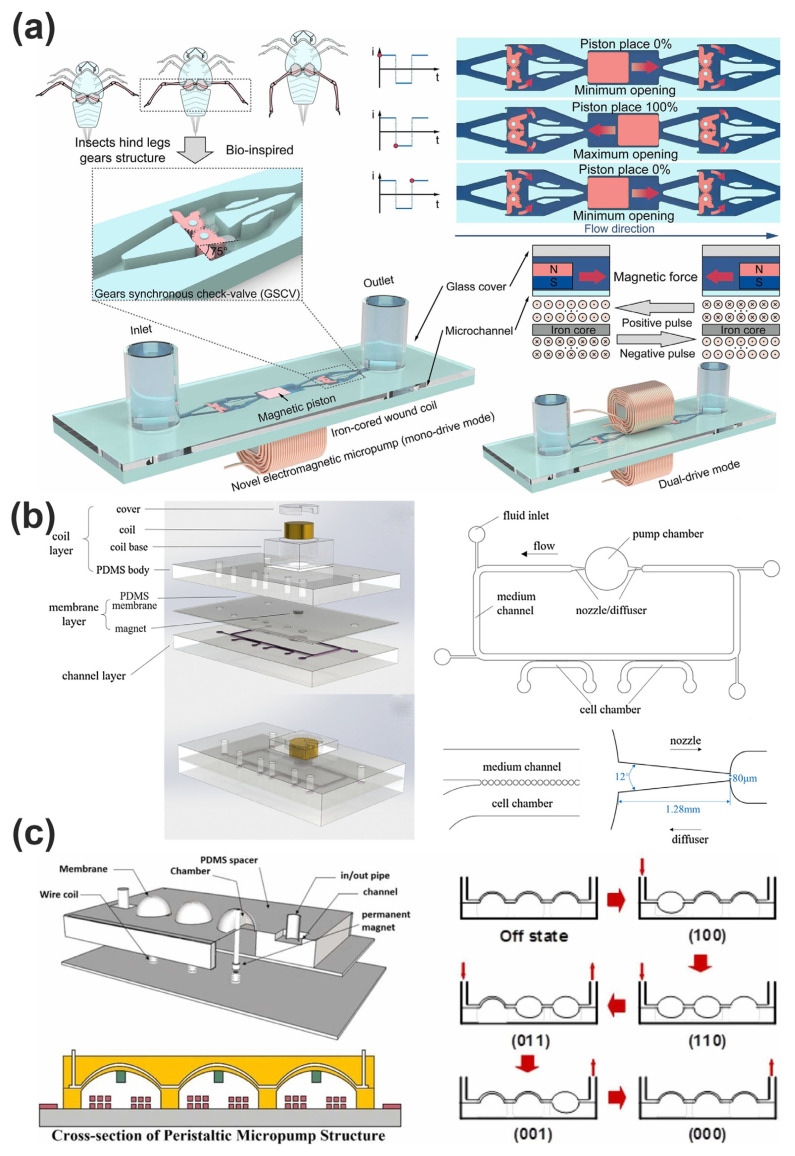
(**a**) Working principles of the micropump and GSCV schematics [108]. Reprinted/adapted with permission from Ref. [108]. 2025, Elsevier. (**b**) The structural design of the micropump and the chip and schematic diagram of the assembled chip. The medium channel and the cell chamber are separated by a microcolumn array [109]. Reprinted/adapted with permission from Ref. [109]. 2025, Elsevier. (**c**) Schematic of peristaltic electromagnetic micropump and the 6-Stage pulse sequence of peristaltic pumping mechanism [122]. Reprinted/adapted with permission from Ref. [122]. 2025, Elsevier.

Another prominent application of electromagnetic actuators within microfluidics is in microvalves, which require precise and reliable control to regulate fluid flow effectively. MEMS electromagnetic valves employ actuators that use magnetic attraction or repulsion forces to move membranes or diaphragms, providing rapid and reversible fluid gating [115,116,117]. Recent advances have facilitated improvements in valve sealing performance, response time, and operational reliability, achieved through optimized actuator geometries, coil configurations, and the incorporation of soft magnetic materials. Such advancements enable enhanced flow control precision, critical for applications in biomedical diagnostics, point-of-care testing, and chemical analysis systems [118,119,120]. Li et al. develop a planar gear synchronous valve structure (GSCV) based on the “meshing gear” movement mode of insects shown in Figure 12a. It can realize rapid response and stable opening in the micro-cavity low-pressure environment and effectively overcome the bottleneck of traditional silicon valves, which are rigid and difficult to open, with an average synchronization ratio of more than 90%. By arranging the coils up and down, the magnetic piston bidirectional pulse drive is realized. The proposed micropump can achieve a maximum net flow of 1913.24 μL/min under dual drive mode, and the volume is only 0.62 cm^3^, achieving a high flow density of 3.09 mL min^−1^ cm^−3^. The micropump has successfully achieved efficient driving of deionized water, ethanol, isopropyl alcohol, and other liquids. The micropump’s advantages in flow density and liquid adaptability make it ideal for use in portable analytical instruments, such as biomarker detection and blood glucose analysis chips [108]. Mi et al. present a valveless electromagnetic micropump that can be used in organ chips. Fluid flow is actuated by the vibration of a PDMS membrane through a varying magnetic field shown in Figure 12b. By reducing the volume ofthe coil and magnet, the size of the electromagnetic micropump is minimized and the micropump and the chip can be portably packaged by using a small signal generation module and a dry battery to supply the coil with a square wave, reducing the dependence on external devices. The flow rate was measured to characterize the actuating performance of the micropump. The results indicated that dynamic coculture, which was actuated by the electromagnetic micropump, was beneficial for cell growth and function compared with the static control group [109]. 

Moreover, electromagnetic actuators have found significant utility in the manipulation of droplets within digital microfluidics. Magnetic field-driven droplet actuation, based on ferrofluidic or magnetically responsive droplets, enables precise droplet splitting, merging, transport, and positioning. These magnetic droplet manipulation systems provide inherent advantages over traditional electrowetting-on-dielectric (EWOD) or pressure-driven techniques, including non-contact manipulation, reduced contamination risk, and simplified integration with external magnetic control systems. Advances in actuator design have improved the precision and reproducibility of droplet positioning and volume control, significantly enhancing the performance and applicability of digital microfluidics in high-throughput screening and biochemical assays [121].

Electromagnetic actuation also facilitates efficient fluid mixing in microscale domains, overcoming the inherent difficulty posed by laminar flow regimes that limit mixing efficacy at small scales. MEMS-based electromagnetic micromixers utilize oscillatory or rotational motions induced by magnetic actuators to generate chaotic fluid motion, significantly accelerating the mixing rates within compact microfluidic channels. Despite these advantages, the integration of MEMS electromagnetic actuators within microfluidic components presents several technical challenges, including fabrication complexity, actuator miniaturization constraints, power consumption optimization, and integration compatibility. Ongoing research is addressing these challenges through the development of simplified microfabrication processes, innovative actuator materials, and energy-efficient electromagnetic coil designs. Continued progress in these areas promises to further expand the utility and performance of MEMS electromagnetic actuators within the rapidly evolving field of microfluidics. As shown in Figure 12c, Subandi et al. present a peristaltic electromagnetic micropump driven by dome-shaped membranes which comprises coil components, magneto-mechanical actuators, and a microfluidic system. The actuator membrane is made of polydimethylsiloxane (PDMS), measuring 5 mm in diameter and approximately 100 µm thick, and fabricated using soft lithography techniques to form a normally deflected dome-shaped membrane structure. By applying electrical DC currents ranging from 500 mA to 1000 mA at frequencies up to 10 Hz, the depletion of the membrane was up to 2 mm, causing fluid flow through the microchannel at a flow rate of 8 mL per minute, which revealed that the peristaltic EM micropump can generate a significant fluid flow rate, making it suitable for pumping controlled fluids within a range of up to 10 mL/minute [122].

## 5. Conclusions

Recent advances in MEMS electromagnetic actuators have significantly enhanced their applicability, performance, and integration potential across diverse microsystem domains. The exploration and development of novel fabrication techniques, innovative actuator geometries, and intelligent control strategies have notably expanded their operational capabilities. These advancements have particularly propelled their implementation into sophisticated applications such as adaptive optical devices, precision microrobotics, and microfluidic components, where their distinctive attributes of precise motion control, rapid response, and high force density are critically advantageous.

Despite the remarkable progress, several challenges still need to be addressed to fully unlock the potential of MEMS electromagnetic actuators. One of the foremost challenges is further miniaturization while maintaining high force output and efficiency. As device dimensions continue to shrink, traditional magnetic materials and coil structures may struggle to maintain sufficient magnetic flux density and actuation force. Addressing this issue requires innovations in materials science, such as the exploration of high-permeability nanomaterials, multi-layer coil structures, and advanced 3D microfabrication techniques to achieve more compact yet powerful designs. Another significant challenge lies in efficient power management and thermal dissipation. MEMS electromagnetic actuators, especially those operating at high frequencies or large displacements, can experience significant power consumption and heat generation. Future research should focus on the development of low-loss magnetic materials, energy recovery mechanisms, and thermal optimization strategies. Integration with advanced power management circuits, such as energy-efficient drivers and adaptive power control, could substantially enhance overall efficiency and stability.

Moreover, robust integration with complex microsystems remains a critical hurdle. The seamless incorporation of MEMS electromagnetic actuators with microfluidic networks, optical platforms, and sensor arrays demands not only mechanical compatibility but also electronic and thermal stability. Advanced packaging techniques, wafer-level integration, and the utilization of flexible substrates could enhance reliability and reduce parasitic effects in highly integrated environments.

From an application perspective, emerging technologies such as wearable medical devices, autonomous microrobotics, and IoT microsystems are expected to benefit significantly from the evolution of MEMS electromagnetic actuators. To meet the growing demands of these cutting-edge fields, future designs will need to prioritize multifunctional integration, adaptive control mechanisms, and long-term stability under varying environmental conditions. Additionally, the incorporation of machine learning and artificial intelligence for real-time adaptive control could further elevate their precision and reliability in dynamic and unpredictable scenarios.

In conclusion, while MEMS electromagnetic actuators have made substantial strides, realizing their full potential in next-generation microsystems will require coordinated advancements in materials science, microfabrication, energy management, and system integration. Overcoming these challenges through interdisciplinary research will not only expand their applicability but also set new standards for performance, efficiency, and reliability in microscale actuation technologies.

## Figures and Tables

**Table 1:** Comparison of actuation principles in MEMS devices.

Actuation Principle	Advantages	Disadvantages	Typical Applications
Electromagnetic Actuation	- Large displacement and high output force - Low operating voltage - Good linearity and fast response	- Inductive effects increase power consumption - Requires magnetic materials	Micro motors, Micropumps, Optical scanners, speakers, Micromirror arrays, Microvalves
Piezoelectric Actuation	- High precision and fast response - High energy conversion efficiency - Compact size	- Small displacement, usually in the micron range - Requires high driving voltage - Exhibits hysteresis	Precision positioning, Microactuators, Inkjet printers, Acoustic sensors
Electrostatic Actuation	- Low power consumption - Easily miniaturized and integrated - Fast response	- Low output force - Requires high voltage - Force decreases sharply with distance	Micro relays, Micromirror arrays, RF MEMS switches
Thermal Actuation	- Simple structure - Easy to achieve large displacement	- Slow response speed - High thermal loss - High power consumption	Microvalves, Thermal actuators, Microfluidic devices
